# Role of Standardized Perfusion Value (SPV) in the characterization of Solitary Pulmonary Nodules (SPN)

**DOI:** 10.1186/1471-2318-11-S1-A52

**Published:** 2011-08-24

**Authors:** A Reginelli, M Petrillo, A Porto, F Iacobellis, S Cappabianca, L Brunese, A Rotondo

**Affiliations:** 1 Section of Radiology, Department ‘‘Magrassi-Lanzara,’’ Second University of Naples, Italy; 2 Department of Radiology, Health Science, University of Molise, Campobasso, Italy

## Background

The SPV(standardized perfusion value) is used to compare tissue perfusion with average whole-body perfusion at enhanced MDCT; the SPV is conceptually similar in its derivation of the SUV(standardized uptake value) used to quantify FDG uptake at PET. The aim of this study was to characterize solitary pulmonary nodule (SPN) comparing SPV and SUV value.

## Patients and methods

Twenty nine patients, age range 52-74, with SPN diagnosed with a chest radiography, underwent MDCT and PET and, if necessary, nodule biopsy. The SPV and SUV value were compared with histological features.

## Results

Of the twenty nine patients, 21 underwent CT-guided FNAB with histological analysis and for 8 “wait and watch strategy”was adopted. Seventeen patients showed malignant SPN and four patients had benignant SPN at CT-guided FNAB. In patients with definite histology the specificity of SPV was 75% compared to 83% of SUV; the sensitivity (88%), the accuracy (85%) and the positive predictive value (94%) were equal for both. The negative predictive value of SPV and SUV was 60% and 67% respectively. The comparison of specificity, sensitivity and accuracy, between SPV and SUV was 90% (r=0.9), 100% (r=1) and 100% (r=1), respectively.

## Conclusions

The similarities between SPV and SUV suggest that the CT-derived SPV may be useful, not only for distinction of benign and malignant lesions, but also for acquisition of prognostic information and assessment of treatment response, with less exposure of ionizing radiations.

